# Effects of aging on eye movements in the real world

**DOI:** 10.3389/fnhum.2015.00046

**Published:** 2015-02-10

**Authors:** Stefan Dowiasch, Svenja Marx, Wolfgang Einhäuser, Frank Bremmer

**Affiliations:** Department of Neurophysics, Philipps-University MarburgMarburg, Germany

**Keywords:** eye movements, aging, real-world gaze, natural environment, self-motion, saccades, tracking eye-movements

## Abstract

The effects of aging on eye movements are well studied in the laboratory. Increased saccade latencies or decreased smooth-pursuit gain are well established findings. The question remains whether these findings are influenced by the rather untypical environment of a laboratory; that is, whether or not they transfer to the real world. We measured 34 healthy participants between the age of 25 and 85 during two everyday tasks in the real world: (I) walking down a hallway with free gaze, (II) visual tracking of an earth-fixed object while walking straight-ahead. Eye movements were recorded with a mobile light-weight eye tracker, the EyeSeeCam (ESC). We find that age significantly influences saccade parameters. With increasing age, saccade frequency, amplitude, peak velocity, and mean velocity are reduced and the velocity/amplitude distribution as well as the velocity profile become less skewed. In contrast to laboratory results on smooth pursuit, we did not find a significant effect of age on tracking eye-movements in the real world. Taken together, age-related eye-movement changes as measured in the laboratory only partly resemble those in the real world. It is well-conceivable that in the real world additional sensory cues, such as head-movement or vestibular signals, may partially compensate for age-related effects, which, according to this view, would be specific to early motion processing. In any case, our results highlight the importance of validity for natural situations when studying the impact of aging on real-life performance.

## Introduction

As we are getting older, the function of the visual system appears to deteriorate. Not only does visual acuity decline in the elderly, but perception (e.g., Billino et al., 2008; Lich and Bremmer, 2014) and eye-movement parameters are also altered (Morgan, 1993). Increased saccadic latencies (Abel et al., 1983; Moschner and Baloh, 1994; Munoz et al., 1998; Klein et al., 2000) and decreased smooth-pursuit gain (Moschner and Baloh, 1994; Ross et al., 1999) are common findings in the literature, while the results for other oculomotor parameters like saccade peak-velocity are inconclusive. Some studies found a decrease during senescence (Warabi et al., 1984; Sharpe and Zackon, 1987; Irving et al., 2006), whereas others could not show a significant correlation of age and saccade peak velocity (Henriksson et al., 1980; Munoz et al., 1998).

In the last decades, the study of eye movements has increased in relevance as gaze serves as an easily accessible, reliable, safe and fast proxy for cognitive processes and as tool to identify possible functional impairments of the brain (Leigh and Zee, 2006). As an example, the measurement of saccade amplitude and velocity offers an indication of the functionality of the saccade generating circuitry in the brainstem (Sparks, 2002). Certain eye-movement characteristics may extend the knowledge of the mechanism underlying some neurological and psychiatric diseases (Gooding and Basso, 2008; Pinkhardt et al., 2008; Marx et al., 2012; Dowiasch et al., 2014), and might in the long-run, support diagnosis in the clinical routine.

Self-motion through an environment induces one of the most fundamental causes for differences between eye movements in the laboratory and the real world. For example, during walking, the eye-movement system encounters distinct demands as compared to sitting still in the laboratory, which is reflected in qualitatively different oculomotor behavior (‘t Hart et al., 2009; ‘t Hart and Einhäuser, 2012). For example, keeping the eyes on a target that is stationary in the world turns from a mere fixation in the laboratory into a tracking eye-movement during self-motion (Niemann et al., 1999), since the projection of every location in our visual field moves across the retina. Likewise, smooth-pursuit eye-movements as performed in the laboratory are often accompanied by head movements and vestibular-ocular reflexes during free real-world movement. Therefore, these eye movements have to integrate self-motion information in order to operate optimally. At the cortical level, this leads to a massive involvement of areas of the dorsal pathway where the processing of self-motion signals primarily takes place (Bremmer et al., 2000). Especially areas like the ventral intraparietal area (VIP; Bremmer et al., 2001, 2002a; Britten, 2008; Wall and Smith, 2008; Chen et al., 2011) and the medial superior temporal area (MST; Duffy and Wurtz, 1991; Bremmer et al., 1999; Gu et al., 2008; Pitzalis et al., 2013) get activated not only by visual but also by vestibular self-motion signals.

Despite the increasing interest in real-word eye-tracking and despite the abundance of literature on how eye movements in the laboratory are affected in healthy aging, to the best of our knowledge, no study has addressed the effects of healthy aging on real-world eye-movement behavior. Such a transfer to the real world, however, seems particularly important, as an increasing number of studies on eye movements in real-world environments and during everyday tasks (e.g., Land et al., 1999; Hayhoe and Ballard, 2005) raise substantial doubt as to whether results from the laboratory can be directly transferred to real-world scenarios (‘t Hart et al., 2009; Foulsham et al., 2011; Tatler et al., 2011; ‘t Hart and Einhäuser, 2012). Since these studies have typically been performed with small samples and within a homogenous age group, they left possibly existing effects of age on real-world eye-movement behavior so far unaddressed. Patient studies on oculomotor deficiencies in disease, in turn, typically include a set of age-matched healthy controls and often span a wider age range, but do not typically assess the factor age explicitly. In this study, we draw on such control data from earlier patient studies to close the gap and test if and if so to what extent age relate to eye movements in a comparably unconstrained real-world setting. Specifically, we tracked participants’ eye movements while they walked in a corridor either looking around freely or tracking a stationary target on the floor during walking. Since re-inviting the same cohort of participants to laboratory measurements was not feasible, we compared the real-world data in our study to common findings from laboratory studies reported in the literature. It is self-evident that there is no 1-to-1 mapping between such tasks. These limitations notwithstanding, any differences between our results and studies performed in the laboratory may suggest how age-related changes in the healthy brain affect gaze behavior in real-life situations. Such findings would underline the need for addressing real-world tasks to complement laboratory measurements towards a full understanding of the mechanisms underlying oculomotor changes during healthy aging and might point towards new research objectives of future studies.

## Methods

### Subjects

The eye movements of 34 participants (31 male, 3 female) between the age of 25 and 85 (mean = 46y ± 18.5y) were analyzed during two everyday tasks in the real world. All participants were originally recruited as healthy controls for patient studies on eye movements in natural environments (Marx et al., 2012; Dowiasch et al., 2014). Each participant had normal or corrected to normal vision and no history of neurological or psychiatric disease. Two of the tasks in these two studies were identical and are used for the present analysis. Both studies were approved by the local ethics committee and were in accordance with the Declaration of Helsinki. All participants gave their written informed consent.

### Data acquisition

**Figure 1:**
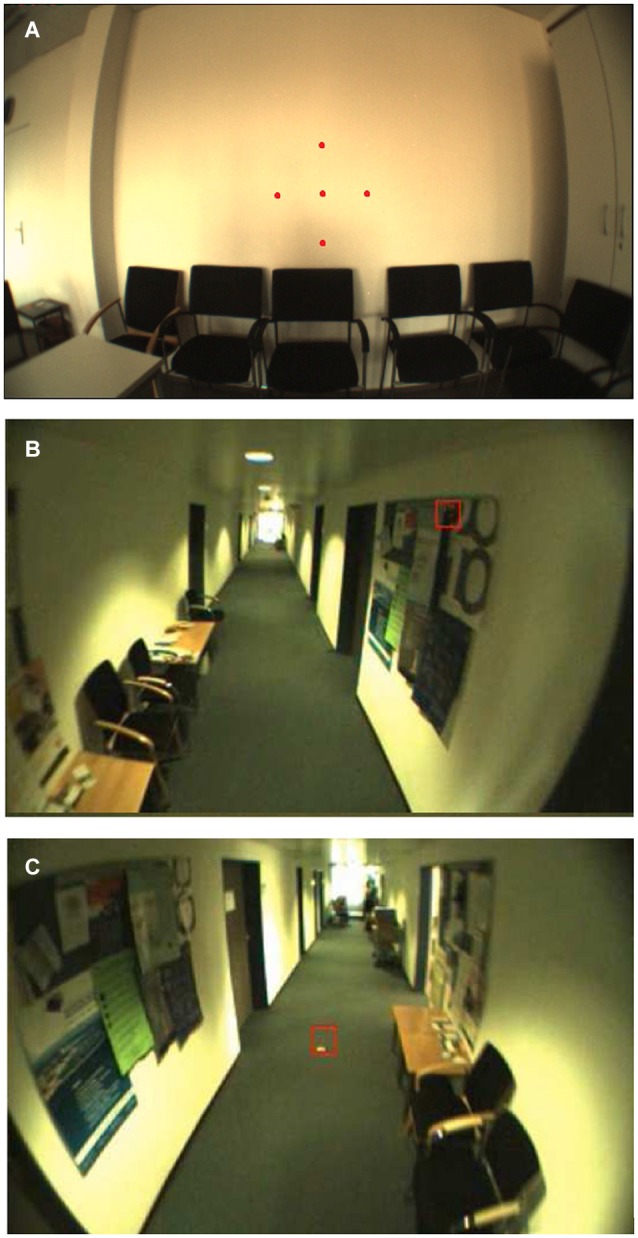
**Illustration of a typical scene during calibration and the two tasks**. Images were taken from the head mounted camera of the ESC. The red square indicates the current gaze position of a participant. **(A)** Calibration: Fixating stationary targets with a fixed distance of 7° as projected by a head-fixed laserpointer of the ESC (enhanced in this figure for visualization). **(B)** Task I: Walking down the hallway with free gaze and **(C)** Task II: visually tracking two stationary targets on the floor while walking straight-ahead.

Binocular eye-in-head movements were recorded with a mobile light-weight eye tracker, the EyeSeeCam (ESC), at a sampling rate of 280 Hz, a spatial resolution of 0.02° and a precision of about 0.1° (Schneider et al., 2009). This allowed us to record and analyze saccadic eye-movements during walking with free gaze reliably. The ESC records a head-centered video with a head-fixed camera and provides a video sequence obtained from a movable camera (GazeCam) which follows the gaze of the participant with a constant latency of about 10 ms (Schneider et al., 2009). Before each measurement the system was calibrated by matching the gaze direction of the subject with the position of 5 predefined targets, which were projected to a plain wall at a distance of 2 m by a head fixed laser pointer (Figure 1A). The mean error threshold for calibration was set to 0.5°. After successful calibration, the participants were asked to perform two different tasks in an indoor environment and to act as they normally would throughout these tasks.

### Behavioral tasks

In the first task participants had to walk along a hallway for about 35 m with free gaze and no additional instruction (Figure 1B). For the second task, they were asked to visually track a stationary orange colored spot with a diameter of 10 cm on the ground (dark green carpet) while walking towards it, starting about 10 m away from the spot (Figure 1C). Each participant was free to choose his/her own walking speed. The duration of the full set of measurements including setup (~1 min), accustoming (at least 2 min; participants indicated when they feel ready to perform the tasks) and calibration (~1 min) of the eye tracker ranged from 5 to 10 min per participant.

Recorded eye-position data and video sequences were analyzed offline using MATLAB 2010b (The MathWorks, Inc., Natick, Massachusetts, USA). In a first step, raw eye-position data was inspected for blinks or other recording artifacts due e.g., to reflections by external light sources. Blinks were classified by the absence of more than 5 samples (18 ms) and eye traces were cleaned for blink artifacts by deleting 8 samples (29 ms) before the start of a blink and 12 samples (43 ms) after a blink. Saccades were detected if eye velocity was higher than 100°/s for at least 3 consecutive samples and if the eyes moved more than 0.5° in this time period. This conservative threshold guaranteed a low false-positive rate for saccade detection, since eye movements during real-life measurements contain extensive dynamics (e.g., due to vestibulo-ocular reflexes or vergence eye movements) and are generally noisier than under controlled laboratory settings. Furthermore, a main-sequence analysis (peak velocity/amplitude) of thus defined saccades was performed by computing the power function fit (*v*_peak_ = *K* * *amplitude ^L^*) and its corresponding 95% confidence interval for each subject (Bahill et al., 1975). All saccades outside this interval were classified as outliers and were not considered for further analysis. Additionally, the saccade velocity profile was characterized by using the q-value, which is defined as the ratio of peak- and mean velocity (*v*_peak_/*v*_mean_; Inchingolo et al., 1987). Finally, saccade amplitude, mean- and peak-velocities were separately analyzed for each of the four cardinal directions (right, left, up, and down). Therefore only saccades with a mean velocity component of more than 100°/s in one of the four directions were considered for analysis to exclude saccades with no specific cardinal direction.

The tracking performance of each participant was quantified by eye-in-head gain values (eye velocity divided by target velocity) and the RMSE (root mean square error) of the retinal target velocity. The rationale for choosing the RMSE, just as for the gain, was its wide use as a global measure of pursuit performance (Smyrnis, 2008) and its good test-retest reliability (Gooding et al., 1994). As a first step in analyzing tracking, all tracking segments were cleaned from saccadic artifacts such as catch-up saccades to analyze the smooth tracking phase only. Since subjects were free to move their eyes in Task I, they typically tracked multiple objects during their way through the hallway (“spontaneous tracking”). In this task only tracking segments longer than 200 ms were considered for further analysis. Accordingly, the reference velocity (target velocity) had to be determined individually for each subject and each eye-movement trajectory. To do so, we computed the optical flow field (Gautama and Van Hulle, 2002) from the head centered video recorded by the ESC. Target velocity was considered the velocity of the image part relative to the head which was tracked by the subjects’ gaze. Due to technical issues in the recording of the head-centered video, (e.g., caused by blurred video or by considerable frame drops), the optic flow field of the recordings from 4 participants (age: 33, 50, 64, and 69) could not be computed reliably. These 4 subjects were excluded from optic-flow analysis and no free-viewing gain was computed.

In Task II the presence of specific tracking targets allowed us to determine target velocity as the temporal derivative of the target position in the head-centered scene. In addition, the GazeCam videos of the ESC could be used to calculate retinal target velocity as the temporal derivative of the target position within this retinocentric framework. The sum of all deviations from the optimal retinal target velocity (0°/s) corresponds to the RMSE. In this task, six participants (ages: 30, 33, 33, 50, 64, 74) did not show a sufficient tracking of the specified target (e.g., they ignored the target at all) and their data were therefore excluded from the evaluation of tracking performance (i.e., tracking-gain as well as RMSE). In addition the RMSE could not be evaluated precisely due to considerable frame drops in the GazeCam-videos of three other participants (ages: 30,32,53).

### Statistics

The analyzed eye-movement parameters (saccade amplitude, saccade peak velocity, saccade mean velocity, and the q-value of the saccade velocity distribution in task I as well as tracking gain and RSME in task II) cannot be expected to follow a normal distribution (Land et al., 1999). Hence we used the non-parametric Mann-Whitney-U-Test (Mann and Whitney, 1947) for all statistical analyses. An alpha-level of 0.05 was used as threshold for significance. We characterized each subject’s respective eye-movement parameter by the median (over saccades and tracking epochs, respectively) rather than by the mean. To calculate significance we performed a median-split analysis, comparing the older half of participants to the younger half. This resulted in a younger group (*n* = 17) with a mean age of 30.1 ± 3.0y and an older group (*n* = 17) with a mean age of 61.8 ± 12.8y. There was no statistical difference in mean age of the younger or older group when comparing the tasks in which participants were excluded and the full group of participants (freeviewing-gain: younger group (*n* = 15): mean age 29.5 ± 2.6y; older group (*n* = 15): mean age 60.2 ± 14.7y; tracking-gain: younger group (*n* = 14): mean age 29.7 ± 3.0y; older group (*n* = 14): mean age 61.6 ± 13.3y; RMSE: younger group (*n* = 12): mean age 29.5 ± 3.2y; older group (*n* = 13): mean age 62.2 ± 13.6y; all *p* > 0.8; U-Test). Additionally the effect size of each result was computed using the “Area under the receiver operating characteristic curve” (AUC or A’) (Bamber, 1975). Similar to d’, AUC can be understood as a measure of overlap of two distributions, with separability being minimal at a value of 0.5 and maximal at 0.0 or 1, respectively (Hentschke and Stüttgen, 2011). The 95% confidence interval for each effect size was calculated analytically (Hanley and McNeil, 1982). Finally, we report Pearson’s linear correlation coefficient with the age of the participants for all eye-movement parameters and its corresponding significance level.

## Results

Thirty-four participants between the age of 25 to 85 performed two different oculomotor tasks in an indoor environment: (I) walking through a hallway with free gaze and (II) visually tracking a stationary object on the ground while walking straight ahead.

### Task I—walking with free gaze

#### Saccade and blink rate

**Figure 2:**
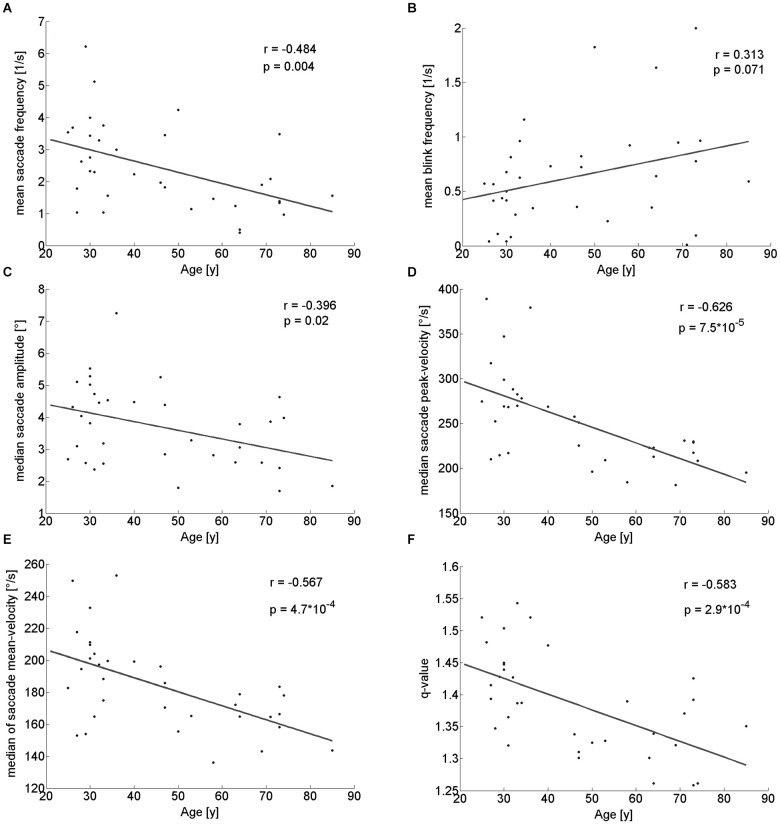
**Pearson correlation of different eye movement parameters with participants’ age**. There is a significant decline with age of saccade frequency **(A)**, saccade amplitude **(C)**, saccade peak- **(D)** and mean-velocity **(E)** and q-value **(F)**. Blink rate trended to be larger in older participants **(B)**.

When walking along a hallway with no specific instructions we neither observed a significant difference in walking time between the younger (mean: 26.5 ± 2.7 s) and the older participants (mean: 24.1 ± 4.8 s; *Z* = 1.54; *p* = 0.124; U-test), nor a correlation of walking time and age (*r*_(32)_ = −0.260; *p* = 0.138). Yet, several eye-movement parameters depended on age. The frequency of saccades showed a significantly decrease in the older participants as compared to the younger (Table 1) and correlated with age (*r*_(32)_ = −0.484; *p* = 0.004; Figure 2A). There was a trend towards an increase in the frequency of eye blinks in older participants, but this trend did not reach statistical significance neither in the median-split analysis (Table 1), nor in the correlation analysis (*r*_(32)_ = 0.313; *p* = 0.07; Figure 2B).

**Table 1:** **Basic eye-movement parameters by task**.

	Eye-movement parameter		U-test				
			Young (SD)	Old (SD)	*Z*-value	*p*-value	Effect-Size (AUC)
Task I (walking with free gaze)	median saccade amplitude [°]		4.14 (1.31)	3.25 (1.07)	1.894	0.058	0.692 [0.513 0.871]
	median saccade peak-velocity [°/s]		285.6 (51.4)	220.2 (24.2)	3.789	0.0002	0.882 [0.764 1.000]
	median saccade mean-velocity [°/s]		199.2 (29.1)	168.3 (17.8)	3.134	0.0017	0.817 [0.671 0.962]
	median q-value		1.40 (0.06)	1.31 (0.06)	3.479	0.0005	0.851 [0.719 0.984]
	main-sequence fit K-value		185.0 (24.7)	154.4 (22.8)	3.169	0.0015	0.820 [0.676 0.965]
	main-sequence fit L-value		0.335 (0.04)	0.359 (0.08)	−0.689	0.491	0.429 [0.235 0.624]
	mean saccade frequency [1/s]		3.03 (1.37)	1.83 (1.04)	2.582	0.010	0.761 [0.598 0.924]
	mean freeviewing-gain		1.31 (0.31)	1.56 (0.29)	−2.224	0.026	0.259 [0.077 0.441]
	mean blink rate [1/s]		0.474 (0.32)	0.801 (0.57)	−1.826	0.068	0.315 [0.135 0.495]
	median saccade peak-velocity [°/s] for certain saccade amplitudes	1°–2° (22.5%)	202.9 (35.1)	187.4 (37.1)	2.032	0.042	0.706 [0.530 0.882]
		2°–3° (11.5%)	244.3 (52.9)	207.9 (28.1)	2.342	0.019	0.737 [0.568 0.906]
		3°–5° (14.7%)	251.6 (28.8)	226.9 (18.6)	2.962	0.003	0.799 [0.648 0.951]
		5°–8° (14.6%)	317.0 (34.8)	283.0 (24.9)	2.997	0.003	0.803 [0.652 0.953]
		8°–15° (15.3%)	413.6 (37.0)	363.9 (41.5)	2.514	0.012	0.855 [0.722 0.988]
Task II (tracking targets)	mean tracking-gain tracking RMSE		0.957 (0.13)	0.865 (0.22)	1.264	0.206	0.643 [0.436 0.850]
			16.99 (6.97)	17.80 (4.90)	−0.680	0.497	0.417 [0.190 0.643]

*Computed eye-movement parameters of the two tasks (Task I and II) and their corresponding statistics. The percentage behind each saccade amplitude window reflects the prevalence of a saccade of that particular amplitude. The distinct analysis of amplitude ranges of more than 15° could not be evaluated reliably, because some participants did not perform sufficient saccades within this domain*.

#### Parameters of individual saccades (amplitude and velocities)

**Figure 3:**
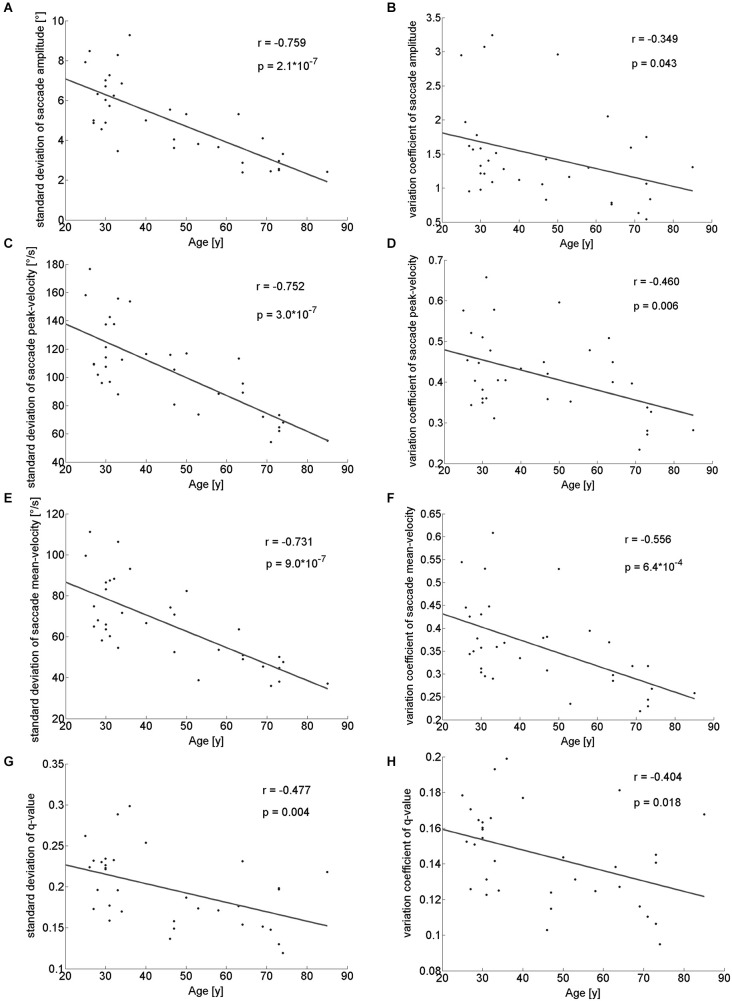
**Standard deviations and variation coefficient of saccade amplitude **(A,B)**, peak- **(C,D)** and mean-velocities **(E,F)** and q-value **(G,H)** in relation to the age of the participants**. There is a clearly negative correlation for these parameters indicating a decreased variability of saccadic performance in older participants.

With respect to the parameters of individual saccades (amplitude and velocity measures), there was a trend towards smaller saccade amplitudes in the older participants, which however, did not reach significance (Table 1). Yet, we found a negative correlation between age and median saccade amplitude (*r*_(32)_ = −0.396; *p* = 0.02; Figure 2C). When analyzing the amplitudes of horizontal and vertical saccades separately only downward and leftward saccades showed a significant decrease in the older group (Table 2). Just as median saccade amplitude, saccade peak and mean velocity were negatively correlated with age (peak-velocity: *r*_(32)_ = −0.626; *p* < 0.001; Figure 2D; mean-velocity: *r*_(32)_ = −0.567; *p* < 0.001; Figure 2E). For those two parameters the median-split analysis showed a clearly significant decrease in the older population (Table 1). This was especially true for saccades in the horizontal direction (Table 2) and also trended to be significantly lower in the older group for downward saccades. Yet, upward saccades did not show any statistical differences between groups for either saccade mean- or peak-velocity (Table 2). Finally, the saccade distribution for older participants was less skewed, as reflected by the significantly lower median q-value (Table 1) and the negative correlation of age with q-value *r*_(32)_ = −0.583; *p* < 0.001; Figure 2F). When analyzing the standard deviations of the saccade parameters (amplitude, mean- and peak-velocities and q-value) in relation to the age of the participants, there was a negative correlation for all of them (saccade amplitude: *r*_(32)_ = −0.759; *p* < 0.001; Figure 3A; saccade peak-velocity: *r*_(32)_ = −0.752; *p* < 0.001; Figure 3C; saccade mean-velocity: *r*_(32)_ = −0.731; *p* < 0.001; Figure 3E; q-value: *r*_(32)_ = −0.477; *p* = 0.004; Figure 3G). This negative correlation remained for the variation coefficient, which serves as the standardized (normalized) measure of dispersion of the four saccadic performance parameters (saccade amplitude: *r*_(32)_ = −0.349; *p* = 0.043; Figure 3B; saccade peak-velocity: *r*_(32)_ = −0.460; *p* = 0.006; Figure 3D; saccade mean-velocity: *r*_(32)_ = −0.556; *p* < 0.001; Figure 3F; q-value: *r*_(32)_ = −0.404; *p* = 0.018; Figure 3H).

**Table 2:** **Saccade mean- and peak-velocity examined separately for each direction**.

	Eye-movement parameter		U-test				
			Young (SD)	Old (SD)	*Z*-value	*p*-value	Effect-size (AUC)
Task I (walking with free gaze)	median saccade amplitude [°] for saccades	Upward	4.39 (1.46)	3.50 (1.69)	1.274	0.203	0.669 [0.484 0.855]
		Downward	5.46 (2.68)	3.79 (1.65)	1.963	0.050	0.699 [0.521 0.877]
		Leftward	7.26 (2.57)	4.40 (1.32)	3.582	0.0003	0.862 [0.734 0.990]
		Rightward	6.66 (2.29)	5.14 (2.21)	1.688	0.092	0.671 [0.489 0.854]
	median saccade mean-velocity [°/s] for saccades	Upward	147.9 (15.4)	139.8 (14.8)	0.861	0.389	0.625 [0.433 0.817]
		Downward	164.9 (32.1)	138.0 (16.9)	2.721	0.007	0.775 [0.616 0.934]
		Leftward	207.9 (41.9)	161.5 (27.2)	3.237	0.001	0.827 [0.685 0.969]
		Rightward	197.1 (32.0)	169.1 (29.3)	2.342	0.019	0.737 [0.568 0.906]
	median saccade peak-velocity [°/s] for saccades	Upward	174.8 (24.5)	170.3 (25.4)	0.241	0.810	0.559 [0.361 0.757]
		Downward	189.1 (45.5)	166.1 (21.2)	1.722	0.090	0.675 [0.493 0.857]
		Leftward	267.0 (55.4)	201.2 (38.2)	3.444	0.0006	0.848 [0.714 0.982]
		Rightward	253.4 (47.2)	199.7 (38.9)	2.961	0.003	0.799 [0.648 0.951]

*Median saccade amplitude, mean- and peak-velocity of the two groups during the first task and their corresponding statistics. Only saccades with a higher mean-velocity of more than 100° for each direction were analyzed to exclude saccades with an unspecific direction*.

#### Main sequence

**Figure 4:**
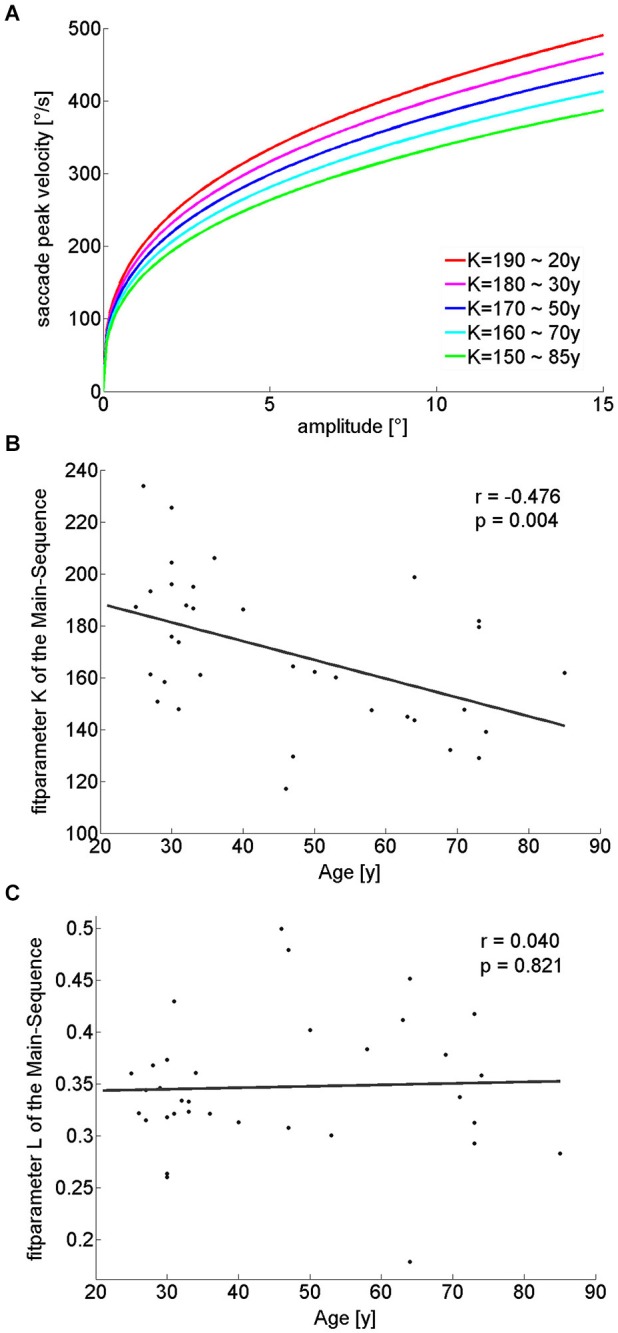
**Illustration of Main-sequence power-function fits for different K-values, while the exponent (“L”) remains unchanged (A), each K-value relates to a different age cohort, as extracted from the linear regression of K-value over age (B)**. L-values were constant at 0.35 for all fits **(C)**. With increasing age, smaller K-values **(B)** lead to decreasing slopes of the power functions and therefore smaller saccade peak-velocities for the same amplitudes when L-values are constant **(C)**.

Saccade amplitudes and velocities are not independent from each other, but coupled through the so-called main sequence. When fitting a power function to the main sequence (see Section Methods) for these amplitude ranges (Figure 4A), the exponent of the power function (“L”), showed no significant difference between the groups (Table 1) or correlation with age (*r*_(32)_ = 0.04; *p* = 0.821; Figure 4C). On the other hand, the fit parameter K, which corresponds to the rise of the power function when the exponent is set, was significantly smaller in older participants (Table 1) and negatively correlated with age (*r*_(32)_ = −0.476; *p* = 0.004; Figure 4B). In addition, the analysis of saccade peak-velocity within certain amplitude ranges showed a significantly smaller peak-velocity in the older participant group for all analyzed amplitudes (Table 1). In sum, nearly all saccade parameters are affected by age, but the general shape of the main sequence remains remarkably unaffected.

#### Spontaneous tracking

**Figure 5:**
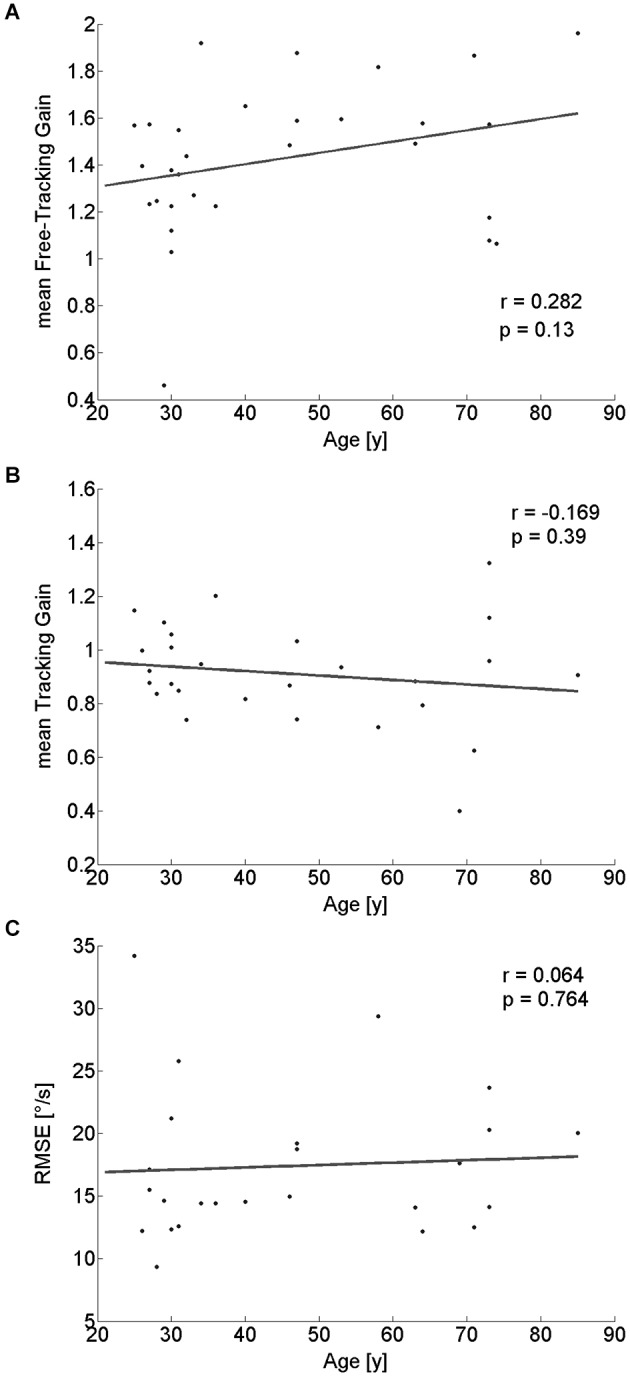
**Pearson correlation of tracking-performance parameters with participants’ age**. Neither tracking gain during free exploration **(A)**, nor gain **(B)** or RMSE **(C)** of the tracking eye-movement of a specific target showed a significant correlation with age.

During the free exploration of task I, there were periods in which participants spontaneously tracked a target during walking. These tracking movements had an average gain above 1 for nearly all (29/30) participants. There was no significant linear dependency on age (*r*_(28)_ = 0.282; *p* = 0.13; Figure 5A), even though a median-split analysis indicated a somewhat higher gain for the older half of observers (Table 1).

### Task II—tracking of a stationary target while walking

For the tracking task, we did not find any significant dependence of tracking gain or tracking performance (as quantified by the RMSE) on age (gain: *r*_(26)_ = −0.169; *p* = 0.39; Figure 5B; RMSE: *r*_(23)_ = 0.064; *p* = 0.76; Figure 5C). Similarly, a median-split analysis did not show any significant difference between the older and the younger half of the participants (Table 1).

## Discussion

In this study we analyzed the age-dependent changes of basic eye-movement parameters in a real-world setting during everyday tasks. Participants were free to move their eyes and head during self-motion. Some of the oculomotor parameters, such as saccade frequency and velocities, showed a significant decline with healthy aging. Others, i.e., tracking performance of an earth-fixed target during self-motion, did not appear to be influenced by age. Accordingly, our saccade data resemble most findings obtained under laboratory conditions. Our findings concerning smooth eye movements, however, challenge the transferability of eye-movement data from the laboratory to the real world.

### Walking with free gaze

In task I, participants had to walk along a hallway with free gaze. A key result was the significant decrease of saccade peak- and mean velocity with age. Previous results on saccade peak velocity in the laboratory were inconsistent and reported both a decline with aging (Warabi et al., 1984; Sharpe and Zackon, 1987; Irving et al., 2006) as well as no significant age dependency (Henriksson et al., 1980; Munoz et al., 1998). While the data of Henriksson et al. (1980) might have had too little statistical power to show a significant effect (6–7 participants per age-group and about 10 saccades for each amplitude investigated), Munoz et al. (1998) investigated only saccades of an amplitude of 20°. Others have hypothesized that an amplitude-dependent saturation in saccade peak velocity in older participants might only affect saccade velocities for amplitudes exceeding 20° (Moschner and Baloh, 1994), which could not be confirmed by our results. The main sequence fit-parameters showed a continuous, significant decline of saccade peak velocity with age.

Mean saccade velocity in the elderly has rarely been examined before. Our results showed a clear reduction for older participants, which was also reported by Spooner et al. (1980) and, with a less strong reduction, by Abel et al. (1983). This effect might be explained by the results of Munoz et al. (1998), who found a significant increase in saccade duration in the oldest participants without an accompanying drop in peak velocity for saccades of the same amplitude. This finding implies a change of saccade skewness with age. Indeed, a generally altered saccade velocity profile of the elderly is also suggested by the q-values as observed in our study. Older participants showed significantly smaller q-values, indicating a less curved saccade velocity profile. This is reflected by a less increased saccade peak-velocity as compared to the saccade mean-velocity.

Another interesting aspect is the separately analyzed age-related difference between horizontal and vertical saccades. For vertical saccades, Huaman and Sharpe (1993) previously reported a decrease in the maximal voluntary excursion in the elderly but no significantly different saccade peak velocity. Our results show a different picture, since saccade amplitude and mean- and peak-velocities were significantly smaller in the older group especially in the horizontal plane. In contrast, in the vertical domain, only the downward saccades showed a trend for being smaller in older participants. This might be mainly due to the nature of the hallway, in which the ceiling is comparably uninteresting/uninformative, as objects and other potential targets of exploration were mostly present in the horizontal periphery.

The general decrease of saccade speed could be due to a loss of contractibility (McKelvie et al., 1999) or mechanical efficiency (Clark and Demer, 2002) of the eye muscles while aging. Concerning their neural basis, saccade properties are defined by burst neurons in the paramedian pontine reticular formation and are not under voluntary control (Sparks, 2002). The significantly decreased saccade velocities and the smaller q-values for older participants suggest a generally lower activity and a less marked burstiness of these neurons. While the brainstem itself seems to be unaffected by healthy aging (Brody and Vijayashankar, 1977), these neurons receive input from the frontal eye fields, superior colliculus, parietal cortex, and basal ganglia (Wurtz and Goldberg, 1989; Sparks, 2002; Leigh and Zee, 2006). Reduced function in one of these areas or brain regions in older participants could be responsible for a decreased firing frequency of premotor or motor neurons and therefore the reduced saccade velocities. For example, it has been shown that frontal lobe lesions can lead to a slowing of saccades (Sharpe, 1986). Indeed, there are studies suggesting a prefrontal functional (Fabiani and Friedman, 1997) and structural (West, 1996) decline with aging. Additionally some studies showed a decreased neuronal density (Huttenlocher, 1979) and a loss of cortical gray matter in the elderly (Pfefferbaum et al., 1994), which might also contribute to a decrease in saccade velocity.

The reduction of saccade frequency, amplitude and velocities could be attributed to a more narrow viewing area of elderly people. This is in line with the decreased variability of saccadic performance in older participants, as shown by the negative correlation of standard deviation of saccadic parameters with age in our study. Yet, the negative correlation of the variation coefficient with age shows, that saccade performance is in general less variable in the elderly. A more narrow viewing area in the elderly is also supported by our results on the separately analyzed horizontal and vertical saccades, which show a consistent decrease in saccade amplitude and velocities in the older group, especially for saccades in the horizontal plane. This might be due to higher effort while walking, e.g., more looking on the pathway to avoid obstacles and plan appropriate motor responses (Di Fabio et al., 2003; ‘t Hart and Einhäuser, 2012), or less confidence in exploring while walking due to a higher likelihood and cost of a potential fall (Hadley et al., 1985). This is in line with the structure of the hallway, in which exploration targets were mostly present in the horizontal periphery. Since objects are the dominant driver of fixations (Stoll et al., 2015), less exploration might be the main reason for the significantly decreased horizontal saccade amplitudes and velocities in the older participants. Chapman and Hollands (2006) have shown that older adults looked significantly earlier to targets, and fixated the targets for longer periods than younger adults while walking along a pathway. The authors explained their result as a consequence of age-related decline in general visual function (Morgan, 1993), slowed cognitive processing (Salthouse, 1996) and decline in visuomotor processing (Moschner and Baloh, 1994).

### Tracking eye-movements during self-motion

In our study, the performance of tracking eye-movements while walking showed different results for spontaneous tracking movements (task I) as compared to instructed tracking movements (task II). Active tracking of optic flow elements in the laboratory has been reported to have a gain close to perfect, i.e., 1.0 (Niemann et al., 1999), which was also the case in our study during tracking of a given stationary target. On the other hand, the gain of most participants during spontaneous tracking was greater than 1.0. This result could be due to the fact that objects in the real world, unlike most artificial stimuli and the target used in our study, have a considerable extent. This leads to more eye movements across the object during the tracking and eventually to a higher speed of the eye during tracking. On the other hand, the computation of optic flow fields of real world scenes can be imprecise because of light reflections or plain surfaces (Gautama and Van Hulle, 2002). This might have led to an underestimation of target velocity in this task. Nevertheless, a median-split analysis showed that the gain of the group of older participants was significantly higher as compared to the younger participants. This suggests a generally more imprecise tracking eye-movement of freely-chosen targets during walking in the elderly. One possible explanation could be an age-related, gradually functional decline of the visual-vestibular system. It has been shown in the laboratory that visual influences on the vestibulo-ocular reflex decline in the elderly together with a deterioration of visual following (Paige, 1994). Accordingly, tracking eye-movements in the elderly might get affected due to differing available input signals.

Unlike smooth pursuit in the laboratory, which has been shown to get worse in the elderly as compared to younger adults (Moschner and Baloh, 1994; Ross et al., 1999), visual tracking performance of a given target during self-motion appeared to be unaffected by age in our study. This finding might suggest compensatory mechanisms, e.g., head movements, or additional sensory cues like optic flow or vestibular signals, which help to maintain normal performance. Paige (1994) found an increased likelihood and intensity of circular vection, a psychophysical measure of visual-vestibular interactions, and proposed an enhanced perception of self-motion in the elderly, which might serve as a visual compensation for age-dependent loss of vestibular cues. Accordingly, optic flow information could neuronally be given a stronger weight in the process of eye-movement control during self-motion. Such an enhanced weight of sensory self-motion information could explain the decreased smooth-pursuit gain in the laboratory due to its absence when measuring with a restrained head. On the other hand motion perception and detection of random dot patterns in the laboratory have been shown to deteriorate in the elderly (Tran et al., 1998). Along similar lines, Billino et al. (2008) showed a gradual decrease in the perception of two-dimensional translational motion and biological motion in the elderly. In contrast, heading detection via expanding radial flow fields was stable across the lifespan in this study (Billino et al., 2008). Nevertheless, a recent study of Lich and Bremmer (2014) showed a decreased absolute heading performance in the elderly in a virtual-reality setting. In this study, the authors were able to model their results in a neural network of visual self-motion processing by an age related neuronal cell loss in area MST. Taken together, this suggests an impairment of motion-selective areas in the brain, such as the middle temporal (MT) area (Newsome and Paré, 1988), the MST area (Duffy and Wurtz, 1991; Bremmer et al., 1999) and the VIP area, which is particularly important in decoding global motion and heading information (Bremmer et al., 2002a; Bremmer, 2005; Chen et al., 2011).

The suggested relevance of self-motion processing for oculomotor performance in the real world as compared to laboratory settings is supported by studies in schizophrenia patients. These patients show an impaired smooth pursuit in the laboratory (Holzman et al., 1974; O’Driscoll and Callahan, 2008) but only a subtle change of tracking eye-movement performance in the real world (Dowiasch et al., 2014). The importance of additional sensory signals to the visual system of schizophrenia patients has been shown by a study of Holzman (2000), in which patients performed worse in a velocity discrimination task when additional non-velocity stimulus cues were eliminated. In natural behavior, these additional cues are almost always present and might serve as a support or even substitute to compensate impairments of specific visual functions. The tracking of a moving object in a real-world situation, while participants are not moving but able to move their head, is an interesting issue for future research. Such a paradigm might be more closely linked to smooth pursuit in the laboratory and therefore might bridge the gap between the results in the literature and the real-world data in our study.

Being aware of the numerous differences between our real-world tasks and typical laboratory measurements, our results provide a first step towards analyzing real-world oculomotor behavior in healthy aging. Furthermore, our correlation analysis together with the effect sizes allowed us to examine to what extent age influences different eye-movement parameters in real-world situations. Together with two recently published studies (Marx et al., 2012; Dowiasch et al., 2014) our results highlight the possible advantages of mobile eye tracking as a fast, reliable, objective and easy-to-use tool, especially when investigating clinical populations or the elderly. Identifying the sources of differences and commonalities between laboratory results and real-world data will be an important issue for future research. Especially the multimodal area VIP (Bremmer et al., 2002b; Schlack et al., 2005), for which a functional equivalent has been identified in humans (Bremmer et al., 2001), might play a crucial role in natural contexts by providing and combining additional sensory information. How such key areas in motion processing are related to the changing oculomotor behavior during aging, and how they integrate the rich information available in the real world for gaze control remains an exciting topic for further research. In any case, our present study underlines the need for addressing real-world situations to fully understand the impact of neuronal changes on oculomotor function and motor behavior in general during healthy aging.

## Conflict of interest statement

The authors declare that the research was conducted in the absence of any commercial or financial relationships that could be construed as a potential conflict of interest.
